# Effects of Dual Peroxisome Proliferator-Activated Receptors *α* and *γ* Activation in Two Rat Models of Neuropathic Pain

**DOI:** 10.1155/2019/2630232

**Published:** 2019-04-17

**Authors:** Mohammad Alsalem, Mansour Haddad, Sara A. Aldossary, Heba Kalbouneh, Belal Azab, Aala Dweik, Amer Imraish, Khalid El-Salem

**Affiliations:** ^1^Faculty of Medicine, The University of Jordan, Amman 11942, Jordan; ^2^Faculty of Pharmacy, Philadelphia University, Amman 19392, Jordan; ^3^Faculty of Clinical Pharmacy, King Faisal University, Al-Ahsa 31982, Saudi Arabia; ^4^Faculty of Science, The University of Jordan, Amman 11942, Jordan; ^5^Faculty of Medicine, Jordan University of Science and Technology, Irbid 22110, Jordan

## Abstract

Neuropathic pain is a growing healthcare problem causing a global burden. Currently used analgesics such as opioids are associated with adverse effects; urging the need for safer alternatives. Here we aimed to investigate the potential analgesic effects of tesaglitazar; dual peroxisome proliferator-activated receptors *α* and *γ* (PPAR*α* and *γ*) agonist in rat models of neuropathic pain. This study also aimed to investigate the modulation of the transient receptor potential vanilloid 1 (TRPV1) receptor activity by tesaglitazar which could provide a potential mechanism that underlie tesaglitazar antinociceptive effects. Von Frey filaments were used to determine the paw withdrawal threshold (PWT) in adult male Sprague Dawley rats (180-250g) following i.p. injection of streptozotocin (STZ) or cisplatin, which were used as models of neuropathic pain. Antinociceptive effects of tesaglitazar were determined 6 hours after drug administration. Cobalt influx assays in cultured dorsal root ganglia (DRG) neurons were used to study the effects of tesaglitazar preincubation on capsaicin-evoked cobalt influx. Both cisplatin and STZ produced a significant decrease in PWT. The higher dose of tesaglitazar (20*μ*g/kg) significantly restored PWT in both neuropathic pain models (P<0.05). 10*μ*M capsaicin produced a robust cobalt response in DRG neurons. Preincubation of DRG neurones with tesaglitazar 6 hours prior to stimulation with capsaicin significantly reduce capsaicin-evoked cobalt responses in a PPAR*α* and PPAR*γ* dependent fashion (P<0.05). In conclusion, tesaglitazar produced significant analgesic effects in STZ and cisplatin-induced neuropathy, possibly by modulating TRPV1 receptor activity. This may be of potential benefit in clinical practice dealing with peripheral neuropathy.

## 1. Introduction

Peripheral neuropathic pain is a significant pharmaceutical and medical problem. It is usually an undertreated secondary symptom of many diseases including diabetes mellitus and an adverse effect of many chemotherapeutic agents such as cisplatin [1]. Peripheral neuropathic pain is one complication of diabetes and a main dose-limiting adverse effect for cancer patients on many chemotherapeutic agents [2]. It may result from toxic insults, disease states, or nerve injury [3]. Several pharmacological families have been investigated for peripheral neuropathic pain conditions including steroids, opiates, selective serotonin reuptake inhibitor (SSRI), cannabinoids, and the tricyclic antidepressant. Often, those pharmaceutical therapies are not effective or safe and merely supportive [4]. Hence, there is an urgent need to identify new effective and safe therapeutic agents for the treatment of peripheral neuropathic pain.

Animal models of peripheral neuropathic pain have been utilized in preclinical studies to characterize complex mechanisms that underlie and maintain the peripheral neuropathic pain. Of those currently used models are diabetes-induced peripheral neuropathy and chemotherapeutics (in particular Cisplatin)-induced peripheral neuropathy. Those two models could be used to investigate different mechanisms of peripheral neuropathy after an injury to develop novel drugs to alleviate or treat the peripheral neuropathy. Diabetic peripheral neuropathy has been suggested to be a putative consequence of chronic hyperglycemia, dyslipidemia, inflammation, and oxidative stress [5]. Cisplatin-induced peripheral neuropathy is a prevalent type of oncology pain and has traditionally been thought to be a consequence of oxidative stress to the mitochondria that leads to apoptosis [6].

Studies in the last decade suggest that activating the Peroxisome proliferator-activated receptors (PPARs) can alleviate peripheral neuropathic pain, perhaps by their anti-inflammatory effects [7]. PPARs are a family of transcription factors that consists of three isoforms including PPAR*α*, PPAR*β*/*δ* (most commonly identified as PPAR*δ*), and PPAR*γ*, which have various tissue expression distributions including pain neuroaxis and distinct physiological roles including inflammation [8]. The commonly prescribed PPAR receptors agonists are the glitazone which has a high affinity for PPAR*γ* [9] and the fibrates which have a high affinity for PPAR*α* [10]. Several glitazones and fibrates have been evaluated separately for their ability to reduce peripheral neuropathic pain [11, 12]. Those studies highlighted the promise of PPAR*α* and PPAR*γ* agonists to alleviate peripheral neuropathic pain. However, a limited number of studies investigated the usefulness of PPAR*α*/PPAR*γ* dual agonists for ameliorating the peripheral neuropathic pain. In this context, PPAR*α*/PPAR*γ* receptors are attractive targets as they are expressed throughout the pain neuroaxis [13]. Targeting both PPAR*α* and PPAR*γ* isoforms could improve the efficacy and provide a superior drug for peripheral neuropathic pain treatment. Tesaglitazar is a dual PPAR*α*/*γ* agonist [14], previously investigated clinically for its ability to treat type 2 diabetes mellitus [15, 16]. Additionally, it was previously shown to reverse CFA-induced mechanical allodynia in rats significantly [17]. However, the potential value of tesaglitazar in alleviating neuropathic pain and the mechanisms that underlie these antinociceptive effects of it, if any, are poorly understood. Transient receptor potential vanilloid 1 (TRPV1) is a key receptor involved in the development of chronic pain conditions. It was shown to underlie pain hypersensitivity in neuropathic pain [18]. Modulating TRPV1 channel activity was proposed to explain the antinociceptive effects of Palmitoylethanolamine (PEA), a PPAR*α* agonist [19–21]. The involvement of both TRPV1 and PPAR*γ* receptors in mediating PEA antinociceptive effect was also reported [22]. These studies indicated a functional and biochemical crosstalk between PPAR receptors and TRPV1 channels. On these bases this study aimed to investigate the therapeutic potential of tesaglitazar in two models of neuropathic pain, Cisplatin-induced peripheral neuropathy and diabetes-induced peripheral neuropathy, and to explore if these effects are dependent on the long-term changes that occur by reducing blood glucose levels. Further aim has been to explore the mechanisms that underlie this action, focusing the attention on the possible interaction with TRPV1 receptor.

## 2. Materials and Methods

### 2.1. Animals

Adult male Sprague Dawley rats (180-250g, Jordan University of Science and Technology laboratories) were used for behavioral experiments. Rats were group housed in the Animal House Unit (The University of Jordan), and free accessibility of food and water was insured throughout all experiments. All procedures were done during the animals' light cycle. Experiments were carried out in accordance with the Animal (Scientific Procedures) Act 1986, International Association for the Study of Pain guidelines and the Scientific Research Committee at the University of Jordan.

### 2.2. Induction of Diabetic Neuropathy Pain Model

In order to induce diabetes, animals received intraperitoneal (i.p.) injections of STZ (35 mg/kg, Tocris Bioscience, UK) or vehicle (0.1M citrate buffer pH 4.6) under general anesthesia with isoflurane inhalation. Body weight, blood glucose levels, and mechanical allodynia were measured before the injections and weekly after that. Diabetes was confirmed by assaying the glucose levels in blood obtained from the tail vein using Accu-Check Performa (Roche, Germany). Rats with glucose levels greater than 200 mg/dl 3 days after STZ injection were considered diabetic.

### 2.3. Induction of Chemotherapy-Induced Neuropathy

Rats were treated with daily intraperitoneal (i.p.) injection of 2.3 mg/kg cisplatin (Sigma-Aldrich) or vehicle (0.9% saline) for 5 days, followed by 5 days of rest, for two cycles. Total cumulative doses of 23 mg/kg cisplatin over a total of ten injections were used. Mechanical allodynia was measured before the injections and at the end of each treatment cycles at days 11 and 21.

### 2.4. Assessment of Mechanical Allodynia

The von Frey filament test was used to measure sensitivity to a punctuate pressure stimulus. Rats were placed in a plastic cage with a wire mesh bottom which allowed full access to the paws. Behavioral accommodation was allowed for at least 25min until cage exploration, and major grooming activities ceased. Subsequently, von Frey filaments (2-15g, with logarithmically incremental stiffness; Bioseb, Vitrolles, France) were applied to the mid-plantar aspect of the left hind paw using the “up-down” method to determine the withdrawal threshold; the von Frey hair was presented perpendicular to the plantar surface and held for approximately 6-8sec [23, 24]. Data are expressed as means ± SEM of paw withdrawal threshold (PWT) in grams.

### 2.5. Pharmacological Treatments

To assess the effects of tesaglitazar on the development of STZ-induced changes in mechanical hind paw withdrawal thresholds. Tesaglitazar (2, 10, or 20*μ*g/kg, Tocris Bioscience, UK) or vehicle (3% tween 20 in saline) was injected i.p. at week 4 after STZ injection, when mechanical allodynia was fully developed. The nociceptive test was performed at 6 hours after tesaglitazar administration. On the other hand, to evaluate the effects of tesaglitazar on the development of chemotherapy-induced neuropathic pain, tesaglitazar (20*μ*g/kg) or vehicle (3% tween 20 in saline) was injected i.p. at the end of the cisplatin treatment cycles. In all behavioral experiments, the observer was blinded to the treatments.

### 2.6. Isolation and Culture of Dorsal Root Ganglia (DRGs) Neurons

Male Sprague Dawley rats (400 g) were decapitated to extract all the DRGs (cervical, thoracic, lumbar, and sacral). The neurons were incubated in Dulbecco's Modified Eagle Medium-F12 (DMEM-F12) containing 0.25% collagenase/30 min/37°C followed by 7 min incubation with 0.25% trypsin in DMEM-F12 media containing 1% HEPES buffer and penicillin/streptomycin (1:200). The neurons were triturated 50 times with normal Pasteur pipettes then 50 times with thin-flame polished pipettes. The neurons were then centrifuged at 400 g for 20 min followed by resuspension of the pellet in media containing 10% heat inactivated fetal bovine serum (FBS). After that, the neurons were triturated again with the thin-flame polished pipettes (50 times), filtered with a cell strainer, and cultured in 96 well-plates for 24 hr at 37°C, 5% CO2.

### 2.7. Cobalt Uptake Assay

After 24 hours, the neurons were treated with different concentrations of capsaicin (100 nm, 1 *μ*M or 10 *μ*M) in 5 mM cobalt chloride for 2 min to choose the concentration of capsaicin for the subsequent experiments. In the control group, only cobalt chloride was added. The cells were then lysed with lysis buffer (20 mM Tris, 150 mM NaCl, and 1% tween 80) without chelating agents. After that, 2-mercaptoethanol (5%) was added to each well, mixed and incubated for few minutes. The color complex was detected by spectrophotometer at 475 nm absorbance. Other wells received capsazepine (10 *μ*M) for 4 minutes prior to 10 *μ*M capsaicin (the chosen concentration in the experiment) to confirm that capsaicin-evoked cobalt influx is TRPV1 mediated. 2 *μ*M tesaglitazar plus 10 *μ*M GW 6471, a selective PPAR*α* antagonist, or 2 *μ*M tesaglitazar plus 10 *μ*M GW 9662, a selective PPAR*γ* antagonist, was applied 6 hours before capsaicin (10 *μ*M) application to assess the effects of tesaglitazar on capsaicin-evoked cobalt influx in DRGs and evaluate the role of PPAR*α* and *γ* (respectively) in mediating theses effects.

### 2.8. Data Analysis

Mechanical allodynia data are presented as means ± SEM of PWT in grams. Blood glucose levels are presented as means ± SEM in mg/dL units. Body weight data are presented as means ± SEM in grams. Two-way ANOVA analysis of variance used with time and treatment are the main factors. Significant ANOVA (p≤0.05) was followed by Holm-Sidak post hoc test. Also, Student's* t*-test or one-way ANOVA test followed by Dunnett's post hoc was used as appropriate. For cobalt uptake assay, data were presented as percentage of cobalt absorbance in treated wells compared to vehicle-treated wells and presented as means ± SEM of % cobalt influx. Statistical analysis used one-way ANOVA test followed by Dunnett's post hoc or Tukey's post hoc as appropriate. All tests were performed using Graph Pad statistical program (Prism 6, San Diego, CA, USA).

## 3. Results

### 3.1. Effects of Tesaglitazar on Diabetic Neuropathy Pain

**Figure 1:**
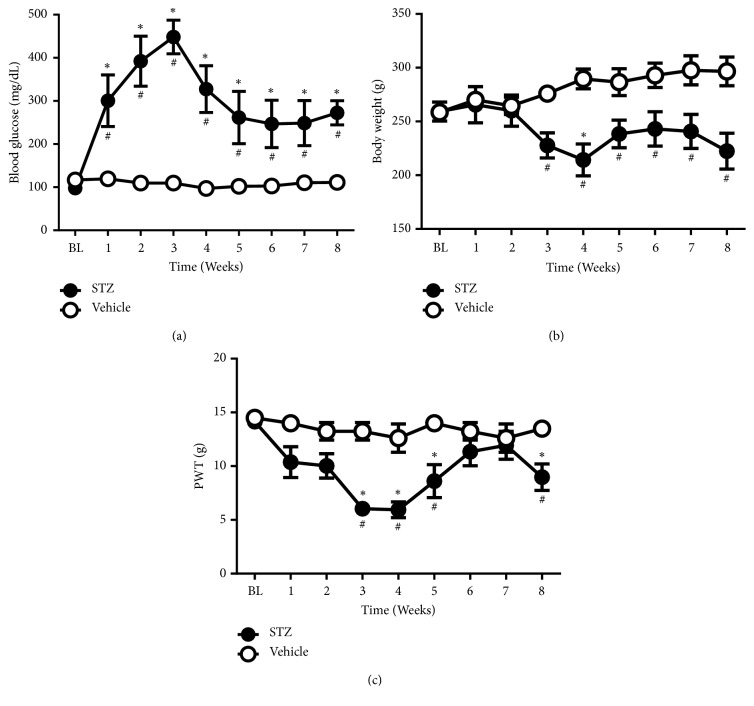
Effects of i.p. injection of STZ on (a) blood glucose, (b) body weight in grams, and (c) mechanical PWT. Two-way ANOVA revealed the following results: (a) significant main effect of treatment [F(1,11)=20.46; p<0.0001], significant main effect of time [F(8,88)=6.535; p<0.0001], and significant main treatment X time interaction [F(8,88)=6.988; p<0.0001]. (b) Significant main effect of treatment [F(1,11)=9.151; p<0.05], no significant main effect of time [F(8,88)=0.9633; p=0.4669], and significant main treatment X time interaction [F(8,88)=5.027; p<0.0001]. (c) Significant main effect of treatment [F(1,11)=43.15; p<0.001], significant main effect of time [F(8,88)=4.289; p<0.01], and significant main treatment X time interaction [F(8,88)=2.864; p<0.001]. # indicates significant difference between groups. *∗* indicates significant difference versus week 0 (BL) within group. All data represent mean ± SEM of 6 rats. (PWT = paw withdrawal threshold in grams).

After STZ treatment, blood glucose levels were significantly elevated by the first week and remained elevated for the course of the study compared to vehicle-injected rats (Figure 1(a)). As the disease progressed, the body weights of control animals increased, whereas the body weights of STZ-treated animals decreased steadily (Figure 1(b)). Alongside these changes in weight and blood glucose levels, hind paw mechanical withdrawal thresholds were significantly lowered in the STZ-treated rats compared to the vehicle-treated rats (Figure 1(c)), which is indicating the development of overt pain behavior.

**Figure 2:**
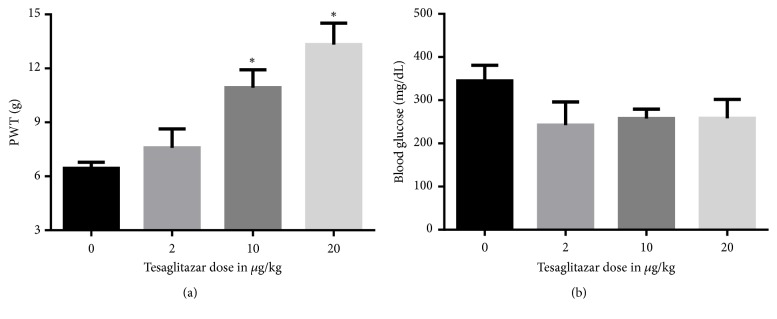
Antinociceptive effects of tesaglitazar on diabetic neuropathy pain. (a) Effects of i.p. injection of tesaglitazar (2, 10, or 20*μ*g/kg) or vehicle on STZ-induced changes in PWT. Data are expressed as mean ± SEM of PWT in grams. (b) Effects of intraperitoneal injection of tesaglitazar (2, 10, or 20*μ*g/kg) or vehicle on STZ-induced changes in blood glucose levels. Data are expressed as mean ± SEM of blood glucose in mg/dL. Rats received i.p. injection of (2, 10, or 20*μ*g/kg) or vehicle at week 4 after STZ injection. Data are analyzed using one-way ANOVA test followed by Dunnett's* post hoc*; all doses were compared to STZ/vehicle (*∗*P<0.05, n=5 rats per group).

At week 4 after STZ injection, when PWT was significantly reduced, tesaglitazar (2, 10, or 20*μ*g/kg) was injected i.p. and antinociceptive properties of tesaglitazar were evaluated 6 hours after drug administration. The higher two doses of tesaglitazar (10 and 20*μ*g/kg) significantly restored PWT (Figure 2(a)). Notably, none of the tesaglitazar doses used in this study has a significant effect on blood glucose levels 6 hours after drug administration (Figure 2(b)).

### 3.2. Effects of Tesaglitazar on Chemotherapy-Induced Neuropathy Pain

**Figure 3:**
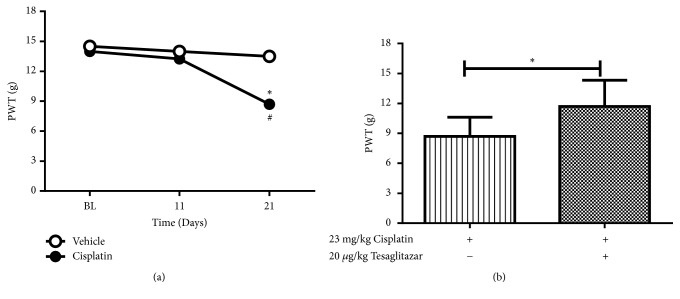
Antinociceptive effects of tesaglitazar on cisplatin-induced neuropathic pain. (a) Effects of i.p. injection of cisplatin (accumulative dose of 23mg/kg) or vehicle on mechanical PWT. Two-way ANOVA revealed the following results: Significant main effect of treatment [F(1,10)=12.29; p<0.01], significant main effect of time [F(2,20)=12.02; p<0.001], and significant main treatment X time interaction [F(2,20)=6.269; p<0.01].# indicates significant difference between groups. *∗* indicates significant difference versus day 0 (BL) within group. (b) Effects of i.p. injection of tesaglitazar (20*μ*g/kg) or vehicle on cisplatin-induced changes in PWT. Data are expressed as mean ± SEM of PWT in grams. Rats received i.p. injection of tesaglitazar (20*μ*g/kg) or vehicle at the end of the cisplatin treatment cycles (day 21). Data are analyzed using Student's* t*-test (*∗*P<0.05, n=6 rats per group).

The findings obtained from this study are suggesting that tesaglitazar's antinociceptive effects are independent of the slower and longer-term cellular and molecular changes that are brought into play by reducing blood glucose levels. To confirm this hypothesis, we evaluated the antinociceptive effects of tesaglitazar on a different model of neuropathic pain (Cisplatin-induced neuropathy pain). After the first treatment cycle, cisplatin-treated rats had no significant reduction in the PWT compared to vehicle-treated controls. However, after the second treatment cycle, cisplatin-treated rats had a significant reduction in the PWT compared to the vehicle-treated group (8.69 ± 0.86 versus 13.49 ± 0.74, *∗P *< 0.05, two-way ANOVA, Figure 3(a)), suggesting the development of apparent pain behavior. In order to evaluate the effects of tesaglitazar on the development of chemotherapy-induced neuropathic pain, 20*μ*g/kg tesaglitazar was injected i.p. at the end of the cisplatin treatment cycles (day 21). Tesaglitazar significantly reversed mechanical PWT 6 hours afterdrug administration (Figure 3(b)).

### 3.3. Effects of Tesaglitazar on Capsaicin-Evoked Cobalt Uptake

**Figure 4:**
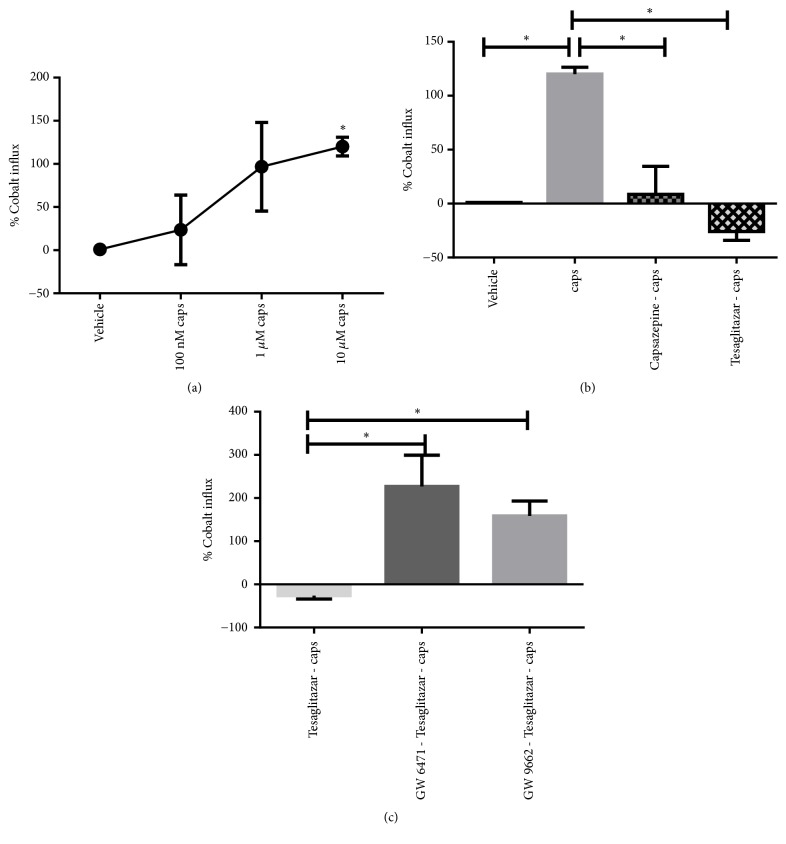
Cobalt uptake experiments: (a) Capsaicin (100 nM, 1*μ*M, and 10 *μ*M) produced a concentration-dependent increase in cobalt uptake in cultured DRG cells. Data are analyzed using one-way ANOVA test followed by Dunnett's* post hoc*; all doses were compared to vehicle (*∗*P<0.05, n=3 wells per treatment). (b) Both capsazepine (10 *μ*M) and tesaglitazar (2 *μ*M) significantly inhibited capsaicin-evoked cobalt uptake in DRGs. Data are analyzed using one-way ANOVA test followed by Tukey's* post hoc*, (*∗*P<0.05, n=3 wells per treatment). (c) GW6471 (PPAR*α* antagonist) or GW9662 (PPAR*γ* antagonist) significantly reduced the inhibitory effects of tesaglitazar on capsaicin-evoked cobalt influx in DRGs. Data are analyzed using one-way ANOVA test followed by Tukey's* post hoc* (*∗*P<0.05, n=3 wells per treatment). All data were presented as percentage of cobalt absorbance in treated wells compared to vehicle-treated wells and presented as means ± SEM of % cobalt influx.

Capsaicin produced a concentration-dependent increase in cobalt uptake in cultured DRG cells; only the highest concentration of capsaicin (10 *μ*M) produced a significant effect (Figure 4(a)). Based on that, 10 *μ*M capsaicin is used for the subsequent experiments. Capsazepine (10 *μ*M) significantly blocks capsaicin-evoked cobalt uptake in DRG neurons (8.63 ± 31.68 versus 120.1 ± 7.68, P value < 0.05, Figure 4(b)) confirming the role of TRPV1 receptor in mediating these effects of capsaicin. Similarly, tesaglitazar (2 *μ*M) significantly inhibits capsaicin-evoked cobalt influx in DRG neurons (-25.89 ± 9.95 versus 120.1 ± 7.68, P value < 0.05, Figure 4(b)). Coadministration of the selective PPAR*α* antagonist GW6471 with tesaglitazar significantly reduced the inhibitory effects of tesaglitazar on capsaicin-evoked cobalt influx in DRGs 227.01 ± 102.38 versus -25.89 ± 7.68, P value < 0.05, Figure 4(c)). Similarly, Coadministration of the selective PPAR*γ* antagonist GW9662 with tesaglitazar significantly reduced the inhibitory effects of tesaglitazar on capsaicin-evoked cobalt influx in DRG cells (158.48 ± 42.39 versus -25.89 ± 7.68, P value < 0.05, Figure 4(c)).

## 4. Discussion

In agreement with previous studies, stable hyperglycemia was observed 3 days after STZ administration in rats. As expected, this diabetogenic action of STZ was accompanied by the development of hyperalgesia in response to mechanical stimulus. This study highlighted the ability of tesaglitazar (10 and 20*μ*g/kg) to relieve pain in STZ-induced neuropathy.

Since tesaglitazar acts as a dual agonist for both PPAR*α* and PPAR*γ* receptors, the involvement of those receptors is likely to mediate its antinociceptive effects. Previous reports revealed that the activation of PPARs decreases the levels of proinflammatory mediators and oxidative stress [25, 26]. Besides, both receptors are expressed in dorsal root ganglia neurons [27, 28], suggesting functional crosstalk. Moreover, the activation of PPARs signaling in both animal and human studies resulted in an improvement of lipid profiles including free fatty acid [29, 30] which is correlated with inflammation in many medical conditions including diabetes [31]. Agonists of the PPAR*γ* and *α* have been reported to regulate inflammation by modulating the production of inflammatory mediators and adhesion molecules. Recently, it has been shown that tesaglitazar significantly reversed the CFA-induced thermal hyperalgesia [17]; our data also showed that tesaglitazar attenuates the cisplatin-induced tactile allodynia. These findings suggest a possible mechanism of action of antinociceptive effect of tesaglitazar by inhibiting the inflammatory signaling.

Most preclinical studies investigating diabetic neuropathy utilized STZ to induce diabetes in rodents [32, 33]. However, other studies used different animal models of diabetes such as Zucker rats and high-fat diet/STZ rats [34, 35]. Although those models of type 2 diabetes can provide useful information about the progression of nerve dysfunction in diabetes [36], our selection of STZ-induced diabetes model, in which hyperglycemia is a distinctive feature throughout the experiment, was based on the simplicity of experimental procedure and cost [37].

The diabetic rat model used in the present study showed latency to onset of neuropathic pain features (3 weeks) and late resolution/remission of allodynia (6-7 weeks). This latency may be attributed to many factors including, firstly, the chronic effect of STZ such as gradual accumulation of glucose and activation of microglia [38], secondly, time-dependent trafficking of targets such as transports or ion channels that may also have delayed onset with prolonged effects and, lastly, short- and long-term cellular and molecular changes that are induced by elevated blood glucose. Regarding the partial remission/resolution, this might occur due to excessive stimulation activity in those targets contributes to a transient tachyphylaxis, tolerance, or desensitization.

Mechanisms underlying neuropathic pain are still poorly understood. Indeed, the supposed mechanisms depend on the type of model used to induce the neuropathic pain. There are many proposed mechanisms in STZ model that are likely to contribute to the development of neuropathic pain, increase in the activity of neurons, and the central sensitization [39], the nerve fiber density of Schwann cells, axons, and intraepidermal might be affected by STZ treatment [40], spinal microglial activation might be increased [41, 42], and the oxidative stress and mitochondrial dysfunction in diabetic neurons might be induced by high blood glucose [43].

The higher two doses of tesaglitazar (10 and 20ug/kg) were needed to significantly relieve the STZ-induced mechanical allodynia in rats after 4 weeks of the induction. This effect of tesaglitazar probably involves the activation of both PPAR*α* and PPAR*γ* receptors. In contrary to previous reports [44, 45], tesaglitazar in the present study did not alter glucose levels in the STZ-treated rats, probably reflecting the low levels of plasma insulin (compared to control rats) in the STZ model. This hypothesis could limit the extent of any improvement in peripheral insulin sensitivity; the STZ rats are insulin deficient, rather than insulin resistant as the rats were unable to secrete any more insulin.

Since the high glucose level has been reported to contribute to the development of diabetic neuropathy, tesaglitazar-induced relief of diabetic neuropathy can be due to an action upon blood glucose level. However, our findings showed that tesaglitazar did not decrease blood glucose level 6 hours after drug administration. This finding is suggesting that tesaglitazar's antinociceptive effects are independent of the slower and longer-term cellular and molecular changes that are brought into play by reducing blood glucose levels. The exact molecular mechanism underlying such effects is still unknown and deserves further investigations. To further investigate this possibility, we evaluated the antinociceptive effects of tesaglitazar on a different model of neuropathic pain. In agreement with previous studies, we have expectedly shown that cisplatin treatment evokes significant tactile allodynia compared to the vehicle-treated controls, suggesting the development of apparent pain behavior. The higher dose tesaglitazar significantly attenuates the cisplatin-induced tactile allodynia. The mechanisms of cisplatin-induced neuropathy are not fully understood; however, studies demonstrate that it is related to axonal damage of peripheral nerves, causing dysfunctional effects in primary afferent fibers that lead to abnormal impulse transmission, nerve hyperexcitability, and pain [46, 47]. Moreover, several studies report that cisplatin-induced neuropathy is mediated by oxidative stress, apoptosis, and inflammation [48]. The inflammatory alterations play a crucial role, especially nuclear factor kappa B (NF-kB) and tumor necrosis factor alpha (TNF-*α*) signaling pathways [49]. Ohta et al. found that the cisplatin enhanced NF-*κβ* phosphorylation significantly through PI3/Akt signaling cascade in ovarian cancer cells [50].

The ability of tesaglitazar to inhibit TRPV1 responses in DRG neurons in a PPAR*α* and PPAR*γ* dependent fashion as revealed by the cobalt influx assays could explain the antinociceptive of tesaglitazar observed* in vivo*. PPAR*α* ligands such as fenofibrate reduced the expression of* cyclooxygenase-2* gene and the production of prostaglandins [51], which in turn are capable of sensitizing TRPV1 channel providing the molecular pathway by which tesaglitazar desensitizes TRPV1 receptor. Anti-inflammatory effects of PPAR*α* have been attributed to the inhibition of the proinflammatory signaling pathways mediated by the transcription-dependent nuclear factor *κβ* (NF-*κβ*) and activated protein-1 (AP-1). This signaling pathway is essential to the development of neuropathic pain as described earlier. Furthermore, tesaglitazar can activate PPAR*α* receptors which in turn inhibit the large and the intermediate conductance K_ca_ channels (BK_ca_ and IK_ca_, respectively) [52]. In agreement with our cobalt influx assay findings, Costa and coworkers (2008) have shown that both PPAR*γ* and TRPV1 receptors mediated the antinociceptive effects of PEA in rat model of neuropathic pain [22].

## 5. Conclusions

This study suggests a new pharmaceutical application of tesaglitazar which exhibits a beneficial property on painful neuropathy characterized by mechanical allodynia and reasoned that tesaglitazar is a potential pharmaceutical agent for treating painful diabetic neuropathy. In the light of the current clinical need for neuropathic pain treatment, this study also provides novel evidence for additional therapeutic potential of PPAR*α*/PPAR*γ* dual activation.

## Data Availability

The data used to support the findings of this study are available from the corresponding author upon request.
